# Non-invasive assessment of liver fibrosis through FibroMeter in patients with chronic viral hepatitis B and C

**DOI:** 10.1186/1471-2334-14-S7-P50

**Published:** 2014-10-15

**Authors:** Oana Săndulescu, Anca Streinu-Cercel, Gabriela Ceapraga, Mara Constantinescu, Adrian Streinu-Cercel

**Affiliations:** 1 Carol Davila University of Medicine and Pharmacy, Bucharest, Romania; 2 National Institute for Infectious Diseases "Prof. Dr. Matei Balş", Bucharest, Romania

## Background

Since the advent of non-invasive methods for liver fibrosis assessment, liver biopsy has been increasingly replaced with liver stiffness measurements or with computed scores based on serum biomarkers, particularly for monitoring viral infections such as HBV [1] or HCV [2,3].

## Methods

We performed a screening study using FibroMeter (Echosens, Paris) to determine the stage of fibrosis and the necroinflammatory status in a cohort of patients with chronic HBV and HCV infection under surveillance in a tertiary care hospital in Bucharest, Romania.

## Results

We analyzed data from 87 patients, 68 (78.2%) of which had chronic HBV infection and 19 (21.8%), chronic HCV infection. The median age was 44.9±15.0 (range 17-75). The mean body mass index (BMI) was 26.0±3.6 in the HBV group and 26.7±3.2 in the HCV group. Overall, 36 patients (41.4%) had normal BMI, another 36 (41.4%) had a BMI equivalent for overweight status, and 15 (17.2%) had grade I obesity.

The distribution of FibroMeter results was: F0-F1: 8 (9.2%), F1: 2 (2.3%), F1-F2: 43 (49.4%), F2: 11 (12.6%), F2-F3: 8 (9.2%), F3: 7 (8.0%), F3-F4: 8 (9.2%). The distribution of the necroinflammatory activity was: A0-A1: 16 (18.4%), A1-A2: 52 (59.8%), A2-A3: 19 (21.8%). We identified no statistically significant differences between patients with HBV and HCV regarding mean fibrosis scores (p=0.476) or mean necroinflammatory activity scores (p=0.681).

## Conclusion

The patients included in this study had varied ages and characteristics. FibroMeter classified most of them as F1-F2 but descriptive data should be interpreted in clinical context and potential confounding factors should be identified on a case-by-case basis.
